# What socio-demographic factors influence poverty and financial health care access among disabled people in Flanders: a cross-sectional study

**DOI:** 10.1186/2049-3258-72-5

**Published:** 2014-02-12

**Authors:** Margo Adams, Nele Augustyns, Herman Janssens, Bart Vriesacker, Guido Van Hal

**Affiliations:** 1 Antwerp University, Universiteitsplein 1, 2610 Wilrijk, Belgium; 2 Catholic Association for Disabled people, Arthur Goemaerelei 66, 2018 Antwerpen, Belgium

## Abstract

**Background:**

Current literature shows that people with a disability have a lower income than people without a disability. Disabled people often experience difficulties with health care access.

The objective of this study is to assess the current financial situation and poverty rate amongst disabled people in Flanders. Furthermore we wanted to analyze factors that contribute to the risk of poverty and problems with financial health care access in adult people with a disability in Flanders.

**Methods:**

An online and paper survey were constructed and made available through two large organizations for people with different types of disability in Flanders. Descriptive statistics and logistic regression analysis were performed.

**Results:**

In this convenience sample, 20.9% of the 889 respondents live under the poverty threshold. Important contributing factors to the risk of poverty are having children (OR 3.43, 95% CI 2.10-5.59) and a low level of dependence (OR 16.40, 95% CI 6.21-43.28). 25.2% of the respondents did not access health care because of financial shortcomings. A low level of dependence is one important contributing factor (OR 3.16, 95% CI 1.41-6.98) to limited financial health care access.

**Conclusion:**

This research confirms that disability is associated with a higher risk of poverty and impaired financial health care access.

## Background

International research on poverty and health care accessibility among disabled people is scarce [1].

Current literature shows that people with a disability have a lower income than people who are not disabled [1,2]. Furthermore people with impaired access to health care tend to be in worse health and with lower income [3,4]. In Belgium as well as in the EU-25, “too expensive” is the main reason for having perceived unmet medical needs [5]. Disabled people in particular experience difficulty with health care access regardless of their income [1,6]. Having a disability decreases both job and housing opportunities [2,7-9].

A lack of information about the current situation in the Flemish region, combined with 2010 being the ‘European Year for Combating Poverty and Social Exclusion’, provided an opportunity to investigate which socio-demographic factors relate to poverty and financial health care access for disabled people.

The Flemish Region comprises 6,251,983 inhabitants (out of almost 11 million Belgian inhabitants; figures for 2010 for the sake of comparability) making it the most populated region of Belgium [10]. Social security policy, public health care and disability matters are executed by federal and regional governments, which makes the public administration system in Belgium quite complex. In Flanders in June 2010 35,298 places in a licensed care institution for disabled people providing accommodation, support and care, were available. Of these, 24,511 places were residential or semi residential care institutions, 10,787 places were reserved for ambulant care and support [11]. Insurance for medical care is compulsory, with a minimum financial contribution, and covers both preventive and curative care. A patient pays the full amount for medical consultations or treatment and receives a receipt. The insurance institution (health insurance fund) will refund part of the costs when the receipt is submitted. Prescribed medicines or drugs are reimbursed through a third payer’s scheme, meaning that a patient with a prescription from a recognized practitioner does not have to pay the full amount at the pharmacy because the reimbursement rates are applied directly. Psychological help is partially reimbursed, but only if it is provided by a psychiatrist. The reimbursement of expenses related to vision aids (glasses or lenses) depends on the patient’s health insurance fund and varies in rate and frequency of reimbursement [12].

In Belgium there are two types of financial compensation for people with a disability^a^. Firstly, an ‘income replacement allowance’ (IRA) is granted to disabled people who are less able or unable to perform work because of physical or mental limitations. Secondly, an ‘integration allowance’ (IA) is granted to disabled people who have an increased level of dependence. The amount for both allowances is influenced by the household type, monthly income and the partner’s income [13].

Some disabled people can benefit from certain important governmental measures to reduce financial barriers in Belgium, such as the RVV-statute, the maximal invoice and the Omnio-statute. A well-defined group of disabled people are entitled to receive an increased reimbursement of medical costs (the so-called RVV-statute, Recht op Verhoogde Verzekeringstegemoetkoming). Those disabled people whose disability is officially recognised by the Federal Public Service Social Security and who are receiving an IRA or IA, get this special status regardless of the level of their income. Disabled people with recognition but without an IRA or IA, are entitled to this status on the condition that their income doesn’t exceed a certain level [14]. Furthermore the maximal invoice (MAF, maximumfactuur) sets a maximum amount of annual expenditure on health care per family that varies according to household income. If medical costs exceed this amount, the additional costs are completely reimbursed [15]. Thirdly, the Omnio-statute gives the right to increased reimbursement of medical costs to all households with a gross taxable income underneath a certain level [16].

The goal of this study was to investigate poverty and financial health care access among disabled people in the Flemish Region, and the association of socio-demographic variables with poverty and financial health care access within this population.

## Methods

In December 2009 a survey was constructed and tested in our study population, i.e. disabled people in the Flemish region. The survey was distributed to all members of the two largest associations for disabled people in Flanders (Catholic Association for Disabled people, CAD, and Association for Persons with a Disability, ADP) as an attachment to their bimonthly member magazines (38,000 members). A version in Braille was also available. Additionally, an online version was available on the website of both the CAD and the ADP until the end of March 2010 [17,18]. The survey was also sent out with the magazine of the Flemish Agency for Persons with a Disability. Lastly, several institutions and services for people with a disability were contacted to disseminate the survey. The sample should be considered a convenience sample. A random 10% sample of the paper versions was checked to troubleshoot for errors, by manually checking the digital data with the paper survey versions. As this is a first study in Flanders we aimed to include as many respondents as possible. Therefore we included people with all types and levels of disability, and no distinction was made in duration of disability during the sampling stage. Hence, this convenience sample defines a heterogenic population.

The survey included four questions concerning health care access from the 2008 Belgian Health Interview Survey (BHIS) [19]. These questions assessed whether respondents or members of their households had postponed necessary medical care for financial reasons in the last twelve months. The questions addressed financial access to a doctor or medical help in general, to dentistry, to psychological help and to visual services. Other topics in our survey were gender, age, having a partner, having children, living situation (living together with someone or not), employment, monthly income and housing situation (in a specialized institution, tenant of a social house/apartment, tenant of a private house/apartment, owner of a house/apartment). Disability-related factors, such as type of disability (mental, physical, auditory, visual, autistic spectrum, psychological, other), recognition of the handicap by the federal government and level of dependence were also assessed. In Belgium, disability is assessed according to level of dependence when someone applies for an integration allowance. The level of dependence is generally determined by a medical assessment, based on a score from zero to three on the following six items: transportation, nutrition, personal hygiene, housekeeping, danger recognition and communication and social interaction. These levels go from one (lowest level of dependence) to five (highest level of dependence) and determine the integration allowance received. We did not apply for approval from an ethics committee, but participants were informed that their responses would be handled anonymously.

The standard poverty threshold from the EU SILC (European Statistics on Income and Living Conditions, 2008) was used to assess poverty. The EU SILC poverty threshold is frequently used in research. The poverty line is set at 60% of the national median equivalent disposable income after social transfers per country for an adult single person. Since family members share expenses, the threshold is raised by a factor of 0.5 for the second and each subsequent person aged 14 and over and by a factor of 0.3 for each of those aged under 14 [20]. The 2008 EU SILC was adjusted for inflation by applying the Belgian consumer price index (CPI) to make it comparable with the monthly income data from 2010. For example, the poverty threshold for a one person household was calculated at a monthly income of €973.67. For a single parent with a child aged under 14, the threshold was €1,265.78, while for a childless couple or a single parent with a child aged 14 or over, it was €1,460.51.

Data were analysed using SPSS® 20.0. Analysis took place in three steps: descriptive statistics (Chi^2^ (χ^2^) for categorical variables, student t-tests for continuous variables), unadjusted and adjusted multivariate logistic regression models were calculated. Descriptive statistics provided a first screening towards influential variables for the logistic regression models. Inclusion of the variables in the final adjusted model was based on a significance level (p value) of p < 0.001. Nevertheless, we have chosen to make an exception concerning two variables. One the one hand, the variable ‘monthly income’ is significantly associated with limited health care access, but it wasn’t included in the logistic model. We have chosen to include only ‘poverty’ as a pure financial variable, because it is adjusted for the household situation. On the other hand, the variable ‘employment status’ is included in the logistic model for conceptual reasons, although it wasn’t significantly associated with both outcome variables. Variables were checked for multicollinearity and no issues were identified. The income of each respondent was compared to the poverty threshold matching his or her family situation to construct the variable ‘living under or above the poverty threshold’. The binary variable ‘impaired financial health care access’ was constructed by adding up all positive answers to the questions on postponing different types of health care. After addition, the indicator was dichotomized (no versus one or more problems with financial health care access).

A multivariate logistic regression analysis was performed. In non-adjusted logistic regression models, independent variables were matched to the two outcome variables (living in poverty and impaired financial health care access) to include the variables that were significantly associated with the outcome variables. The variables were used to construct two adjusted binary logistic regression models for each outcome variable.

## Results

### Characteristics of the respondents

A total of 889 respondents completed the survey without missing values (44.2% female; mean age 45.6, range 19–91). 13.7% of the respondents had children and significantly more women had children (p = 0.003). 26.7% lived in a specialized facility. A majority of the respondents (76.6%) were unemployed or inactive (i.e., retired or unable to work) at the time of the survey. The monthly income category of €1,100 to €1,499 was the most frequently recorded among the respondents (44.8%). See the Additional file 1 for a more detailed description of this convenience sample.

### Factors associated with poverty

Bivariate analysis indicates that one respondent out of five (20.9%; n = 186) was living under the poverty threshold. Women and respondents with children, were more frequently living under the poverty threshold. Living under the poverty threshold was also associated with being less dependent, living together with someone, having a partner and being a social tenant (Table 1).

**Table 1:** Association between poverty threshold (<60% median income, EU SILC) and financial health care access and demographic variables gender, age, dependence level, living alone or together, having a partner, housing situation, having children, monthly income, employment and poverty threshold (n = 889), Flanders, 2010

		**Poverty threshold**	**Financial health care access**			
		**Below**	**At least one problem**			**N**
		**%/mean**		**%/mean**		
**Gender**	Male	15.9	**	19.6	**	496
	Female	27.2		32.3		393
**Age**	Continuous	45.6		47.0		
**Level of dependence**^**1**^	1 (i.e. low)	42.6	**	41.5	**	183
	2	24.9		34.7		225
	3	18.4		23.5		196
	4	7.2		9.4		138
	5 (i.e. high)	4.1		7.5		147
**Living situation**	Alone	10.6	**	19.5	**	518
	Not alone	35.3		33.2		371
**Partner**	Yes	38.9	**	20.5	**	229
	No	14.7		38.9		660
**Housing situation**	Institution	6.3	**	5.1	**	237
	Private tenant	25.9		38.6		189
	Social tenant	27.3		34.4		154
	House owner	25.9		27.8		309
**Having children**	Yes	55.7	**	22.4	**	122
	No	15.4		42.6		767
**Monthly income**	< €700	100.0	**	39.4	**	33
	€700 - €1099	36.2		37.2		207
	€1100 - €1499	12.5		16.5		399
	€1500 - €1899	18.8		29.2		96
	€1900 - €2299	10.4		34.3		67
	€2300 - €2699	8.3		22.2		36
	€2700 - €3099	0.0		28.0		25
	≥ €3100	0.0		7.7		26
**Employment**	Yes	18.3		21.6		208
	No	21.7		26.3		681
**Poverty threshold**	Under	/		46.8	**	665
	Above	/		19.5		224

**p = <0.001.

^1^In Belgium, disabled people are assessed according to categories of dependence. These categories determine the financial allowance received and go from one, i.e. the lowest level of dependence, to five, i.e. the highest level of dependence.

In the unadjusted logistic regression analysis, all variables except age and unemployment showed a predictive relationship with living under the poverty threshold. The model, containing all variables depicted in Table 2, shows that being female, having children, having a low dependence level and living with someone remain important predictors for living under the poverty threshold. Furthermore, unemployment was a significant predictive factor in the multivariate model, although unadjusted analysis did not show a significant relationship. The model for living under the poverty threshold had a pseudo R^2^ of 0.333 (Table 2).

**Table 2:** Unadjusted odds ratios and logistic regression model for living under the poverty threshold (n = 889), Flanders, 2010

			**Unadjusted**			**Model**^**b**^		
	**Categories**	**% or mean**	**OR**	**[95% CI]**	**p**	**OR**	**[95% CI]**	**p**
							**Pseudo R**^**2**^ **= 0.333**	
**Gender**	Male (ref)	55.8						
	Female	44.2	1.97	[1.42-2.74]	**	1.48	[1.01-2.17]	*
**Age**	Continuous	45.6	1.00	[0.98-1.01]		1.00	[0.98-1.01]	
**Level of dependence**^**a**^	Category 5 (ref)	16.5						
	Category 1	20.6	17.45	[7.33-41.58]	**	16.40	[6.21-43.28]	**
	Category 2	25.3	7.79	[3.26-18.61]	**	6.45	[2.49-16.68]	**
	Category 3	22.0	5.29	[2.16-12.92]	**	4.13	[1.60-10.64]	*
	Category 4	15.5	1.84	[0.65-5.19]		1.84	[0.63-5.41]	
**Children**	No children (ref)	86.3						
	At least one child	13.7	6.93	[4.61-10.41]	**	3.42	[2.10-5.59]	**
**Housing situation**	Institution (ref)	26.7						
	Private tenant	21.3	5.18	[2.80-9.59]	**	0.85	[0.40-1.81]	
	Social tenant	17.3	5.55	[2.95-10.44]	**	1.16	[0.55-2.47]	
	House owner	34.8	5.17	[2.89-9.25]	**	0.68	[0.32-1.46]	
**Employment**	Yes (ref)	23.4						
	No	76.6	1.24	[0.83-1.85]		2.64	[1.65-4.21]	**
**Living situation**	Alone (ref)	58.3						
	Not alone	41.7	4.60	[3.23-6.53]	**	2.58	[1.46-4.54]	**
**Partner**	No (ref)	74.2						
	Yes	25.8	3.69	[2.62-5.20]	**	1.52	[0.88-2.64]	

*p = <0.05, **p = <0.001.

^a^In Belgium, disabled people are assessed according to categories of dependence. These categories determine the financial allowance received and go from one, i.e. the lowest level of dependence, to five, i.e. the highest level of dependence.

^b^Model contains all variables from unadjusted analysis.

### Factors associated with financial health care access

25.2% of all respondents did not access health care for financial reasons in the past two months (n = 224). In the past 12 months, 13.4% were unable to acquire vision aids, 12.6% did not access dental care, 11.8% did not consult a doctor and 11.6% did not access mental health care because of financial reasons. Almost half of the respondents living under the poverty threshold (46.8%) had impaired financial health care access (p < 0.001). Furthermore, women, parents, tenants in general, respondents who live with someone, respondents who have a partner and respondents with a low dependence level had more difficulties with financial health care access (Table 1).

For financial health care access, all variables showed a significant predictive relationship except age and unemployment in the unadjusted analysis. Significant predictors in the model were having children, a low level of dependence, not living in a specialized facility and living under the poverty threshold. In this case, unemployment also showed up as a significant predictor in the model, but not in the unadjusted analysis. The regression model had a pseudo R^2^ of 0.256 (Table 3).

**Table 3:** Unadjusted odds ratios and logistic regression model for impaired financial health care access (n = 889), Flanders, 2010

				**Unadjusted**			**Model**^**b**^	
	**Categories**	**% or mean**	**OR**	**[95% CI]**	**p**	**OR**	**[95% CI]**	**p**
							**Pseudo R**^**2**^ **= 0.256**	
**Gender**	Male (ref)	55.8						
	Female	44.2	1.96	[1.45-2.67]	**	1.39	[0.99-1.96]	
**Age**	Continuous	45.6	1.01	[1.00-1.02]		1.01	[1.00-1.02]	
**Level of dependence**^**a**^	Category 5 (ref)	16.5						
	Category 1	20.6	8.78	[4.44-17.35]	**	3.16	[1.43-6.98]	*
	Category 2	25.3	6.56	[3.35-12.86]	**	2.71	[1.27-5.80]	*
	Category 3	22.0	3.79	[1.89-7.62]	**	1.76	[0.82-3.78]	
	Category 4	15.5	1.29	[0.56-2.98]		0.86	[0.36-2.09]	
**Children**	No children (ref)	86.3						
	At least one child	13.7	2.57	[1.73-3.82]	**	1.30	[0.80-2.12]	
**Housing situation**	Institution (ref)	26.7						
	Private tenant	21.3	11.80	[6.16-22.61]	**	5.92	[2.82-12.42]	**
	Social tenant	17.3	9.84	[5.04-19.21]	**	4.88	[2.30-10.35]	**
	House owner	34.8	7.23	[3.84-13.60]	**	3.66	[1.73-7.74]	**
**Employment**	Yes (ref)	23.4						
	No	76.6	1.29	[0.89-1.87]		2.13	[1.41-3.21]	**
**Living situation**	Alone (ref)	58.3						
	Not alone	41.7	2.05	[1.51-2.78]	**	0.80	[0.47-1.34]	
**Partner**	No (ref)	74.2						
	Yes	25.8	2.47	[1.78-3.43]	**	1.64	[0.97-2.77]	
**Poverty threshold**	Above (ref)	79.1						
	Under	20.9	3.63	[2.58-5.12]	**	1.86	[1.23-2.80]	*

*p = <0.05, **p = <0.001.

^a^In Belgium, disabled people are assessed according to categories of dependence. These categories determine the financial allowance received and go from one, i.e. the lowest level of dependence, to five, i.e. the highest level of dependence.

^b^Model contains all variables from unadjusted analysis.

## Discussion

More than one fifth of disabled people in our study live in poverty. This means a larger proportion of our sample population lives in poverty, compared to the Flemish general population (one tenth) and the Belgian general population (one seventh) [21-23]. Nevertheless, these findings are in line with literature findings, showing that disabled people not only frequently have a lower income, but are also more often living under the poverty threshold [1,2,24-27]. Our study may underestimate the actual percentage of disabled people living in poverty because we did not take disability-related expenses into account, which can seriously diminish one’s disposable income [27]. A US study showed that disabled people spend six to seven times more on health care than the general population [28]. The inclusion of health care expenses in future research could give a more accurate view of the poverty rate among disabled people and could possibly aid in identifying appropriate measures to reduce this poverty.

One out of four of the respondents had experienced difficulties with financial health care access. This proportion is larger than in the Flemish general population (one tenth) [19]. Expenses for dental care or vision aids are postponed most often, which is partially in line with literature findings. Previous studies showed that a dentist’s visit is number one of the basic health necessities that is postponed among disabled people [6]. In addition, a US study that used similar questions to those in our study, found that people with a disability postponed their visits to the dentist and to the general practitioner more often than a control group without a disability [29]. In this survey only financial barriers to health care were assessed. Other studies reveal other barriers that could possibly cause impaired health care access, for example waiting lists, impaired mobility, fear and others [5].

Current developments in Flemish policy regarding disabled people include a new initiative to provide a personal standard budget. The budget is meant for people with a disability with a strong need for support in activities of daily living, like personal hygiene, mobility, preparing and consuming food, house maintenance, household tasks and communication [30]. Although it isn’t meant to be used to finance medical expenses, the budget can be used to finance medical expenses, since there are no restrictions in the way it should be spent. In this way, this personal standard budget could help disabled people to improve their financial situation and their financial health care access.

The ministry of Social Affairs and Public Health is developing a legislation concerning improvement of health care access for people with chronic diseases. People with a documented rare disease, people with health care expenses over €300 for eight consecutive trimesters and people with an allowance for high health care expenses are qualified to comply with the legislation. Advantages for those qualified include mandatory application of the third payer’s scheme (as of the 1st of January 2015) for example. The mandatory application of the third payer’s scheme for this selected group of patients could lower their financial barrier to health care, since these patients will not have to pay the full amount for medical consultation. The reimbursement rates are applied directly and the health insurance institution will remunerate the health practitioner. Another advantage of the mandatory application of the third payer’s scheme could be an elimination of embarrassment, because ‘asking for it’ could be a possible barrier. However it could be important to emphasize that disabled people with a high level of dependence will probably benefit most from this measure. It is not clear to which extent this measure will add a benefit for people with a low dependence level and lower health care expenses.

Another recent development is the ministry’s intention to shorten the processing time for implementing reimbursement rates to a maximum of six months and free treatment for a specialized group of patients. Immediate reimbursements could improve the financial status at short notice and lower the financial barrier to medication. Also an obligation for hospitals to transparently show their health care cost to their patients and the possibility for social and health care associations to supply for subsidy for trials try to improve the financial position of disabled people in Flanders [31]. The latter measure can stimulate researchers to extend the current knowledge in this domain.

Furthermore, there was the 2% increase of the income replacing allowance to match the minimal income with the increasing welfare in September 2013 [32].

Female respondents more frequently experience poverty and limited financial health care access, which, according to literature, might be caused by their low income [25]. Female poverty can also be linked with having children, which was also shown to be a significant predictor of being poor. Another important risk factor for poverty and limited financial health care access is having a low dependence level. A possible explanation for this finding is that people who are less dependent receive lower support allowances. Unemployment (i.e., the lack of an employment income) is a risk factor for living under the poverty threshold and impaired financial health care access. Literature shows that disabled people have fewer job opportunities [2,7-9]. These results indicate that the subpopulation of disabled people who are unemployed and who have a low level of dependence have a higher risk for poverty and for difficulty in accessing health care because of financial reasons. Furthermore, this indicates that the current labour market offers limited opportunities for them to change this situation. Future research should examine the unemployment status of this population more specifically.

In this study, living with someone seems to be associated with a higher risk of poverty. This could point to an inadequate adjustment of the level of the allowances according to the family situation.

Several studies have investigated the ‘social gap’ in Belgium. The starting point in these studies is the general population with a focus on socio-economic inequalities in health expectancy [33] or socio-economic differences in the utilisation of health services [34]. One study, comparing populations with different educational levels, showed that differences in the prevalence of disability accounted for at least 66% of the inequality in disability-free life expectancy [35]. In our study, however, the starting point were the disabled people themselves, which opens a new perspective on health (care) inequalities in Belgium.

### Strengths and limitations

With a sample size of 889 respondents, this sample accounts for approximately 1.2% of all Flemish disabled people with an income replacement or integration allowance in 2010 (76,129) [13]. Participation in our study was a priority, leading us to use different channels of recruitment but making it impossible to determine an accurate response rate. By including questions from the Belgian Health Interview Survey, we were able to explore differences and similarities between the study population and the general population. The poverty threshold as defined by the EU SILC is commonly used in other studies and this instrument is the EU reference source for comparative statistics on income distribution and social inclusion at the European level and is also recommended by Eurostat [36]. The close cooperation with the CAD (for the survey construction, implementation and interpretation of the results) makes this study a strong reference for other regional, national and even European studies. However, our study is limited by response bias. Some surveys were completed by proxy (e.g., family, caregiver,…), and we did not ask for details about who filled in the survey. The potential influence of this cannot be assessed but we assume parents, family or caregivers may have a different view of the respondent’s situation.

### Suggestions for future research

We would like future research to focus on possible structural measures in order to decrease poverty and impaired health care access amongst disabled people. In particular we would recommend studies to investigate measures to diminish postponing health care visits. Furthermore some more research regarding medical costs for disabled people in Belgium is necessary. We suggest future research to include the duration of the disability (inborn or acquired disability) and the social background of the disabled respondents. As in this study we focused on financial barriers concerning health care access, we would recommend researchers in this domain to explore other barriers in health care access, for example mobility.

## Conclusion

Poverty, as well as impaired health care access, are problems that people with a disability in Flanders frequently have to face. Current research still lacks comprehensive information on the financial, social and medical situation for this target population. Our study hopes to encourage more researchers to engage in this kind of research and to include people with a disability more often in research on social inequalities and poverty. Future research should not only focus on the causes of the current fragile position of disabled people but should also look for both remediating and preventive solutions for these issues of poverty and financial health care access.

## Endnote

^a^The Flemish Institute for People with a Disability defines ‘disability’ as ‘every long term and significant problem to participate of a person that is due to a combination of functional disorders or impairments to execute activities on the one hand, and contextual factors (individual and environmental) on the other.

## Competing interest

The authors declare that they have no competing interests.

## Authors’ contribution

MA: conception, design, acquisition of data, analysis and interpretation of data, drafting the manuscript. NA: conception, design, acquisition of data, analysis and interpretation of data, drafting the manuscript. HJ: conception, design, acquisition of data, interpretation of data, revising the manuscript critically for important intellectual content. BV: conception, design, analysis and interpretation of data, revising the manuscript critically for important intellectual content. GVH: conception, design, interpretation of data, revising the manuscript critically for important intellectual content. All authors read and approved the final manuscript.

## Supplementary Material

Additional file 1 Description of the population. Click here for file

## References

[B1] Yeo R Moore K Including disabled people in poverty reduction work: “nothing about us, without us” World Dev 2003 72 3 571 579 10.1016/S0305-750X(02)00218-8

[B2] Ruts K Inclusiespiegel Vlaanderen: deelname van personen met een beperking aan de samenleving 2006 Brussel: GRIP vzw

[B3] Allin S Masseria C Unmet need as an indicator of access to health care in Europe The London School of Economics and Political Science. Research note http://ec.europa.eu/social/BlobServlet?docId=4741&langId=en. Accessed October 2013

[B4] Porterfield SL McBride TD The effect of poverty and caregiver education on perceived need and access to health services among children with special health care needs Am J Public Health 2007 72 2 323 329 10.2105/AJPH.2004.055921 17194872 PMC1781389

[B5] Baert K De Norre B Population and Social conditions. Eurostat. Statistics in Focus 24/2009. Perception of health and access to health care in the EU-25 in 2007 2013 http://epp.eurostat.ec.europa.eu/cache/ITY_OFFPUB/KS-SF-09-024/EN/KS-SF-09-024-EN.PDF. Accessed October

[B6] Pezzementi ML Fisher MA Oral health status of people with intellectual disabilities in the southeastern United States J Am Dent Assoc 2005 72 7 903 912 10.14219/jada.archive.2005.0291 16060471

[B7] Devisch F Samoy E Lammertyn F Barrières voor de sociale integratie van personen met een handicap 2000 KUL: Leuven

[B8] Shier M Graham JR Jones ME Barriers to employment as experienced by disabled people: a qualitative analysis in Calgary and Regina, Canada Disabil Soc 2009 72 1 63 75 10.1080/09687590802535485

[B9] Koukouli S Vlachonikolis IG Philalithis A Socio-demographic factors and self-reported functional status: the significance of social support BMC Health Serv Res 2002 72 1 20 10.1186/1472-6963-2-20 12361478 PMC130039

[B10] Eurostat Population on 1 January by age and sex – NUTS 2 regionshttp://appsso.eurostat.ec.europa.eu/nui/show.do?dataset=demo_r_d2jan&lang=en. Accessed June 2013

[B11] VAPH - Zorgregierapport 30 juni 2010 http://www.vaph.be/vlafo/view/nl/464335-Zorgvragen.html. Accessed October 2013

[B12] Social Security – Belgian Government https://www.socialsecurity.be. Accessed June 2013

[B13] Federale Overheidsdienst Sociale Zekerheid Jaaroverzicht 2010 in cijfers Directie-Generaal Personen met een handicap http://handicap.fgov.be/sites/handicap.fgov.be/files/explorer/nl/overzicht-cijfers-2010.pdf. Accessed June 2013

[B14] RIZIV Rechthebbenden op de verhoogde (verzekerings)tegemoetkoming (RVV) http://www.riziv.fgov.be/citizen/nl/medical-cost/SANTH_4_4.htm. Accessed October 2013

[B15] RIZIV De Maximumfactuur (MAF) http://www.riziv.fgov.be/citizen/nl/medical-cost/SANTH_4_3.htm. Accessed October 2013

[B16] RIZIV Omnio-statuut http://www.riziv.fgov.be/citizen/nl/medical-cost/SANTH_4_5.htm. Accessed October 2013

[B17] Katholieke Vereniging Gehandicapten http://www.kvg.be. Accessed June 2013

[B18] Vereniging Personen met een handicap http://www.vfg.be. Accessed June 2013

[B19] Demarest S Toegankelijkheid van Gezondheidszorgen Wetenschappelijk Instituut voor Volksgezondheid https://www.wiv-isp.be/epidemio/epinl/CROSPNL/HISNL/his08nl/r4/3.AC_Toegankelijkheid%20van%20gezondheidszorgen_report_2008_NL.pdf. Accessed June 2013

[B20] Eurostat. European Commission People at risk of poverty or social exclusion http://epp.eurostat.ec.europa.eu/statistics_explained/index.php/People_at_risk_of_poverty_or_social_exclusion#Context. Accessed October 2013

[B21] Belgian Ministry of Economic Affairs Data sources http://bestat.economie.fgov.be/BeStat/BeStatMultidimensionalAnalysis;jsessionid =0000zMb1k7T-IkrCweLKnnJIQLa:13rvc52ar. Accessed June 2013

[B22] Eurostat GDP per capita in PPS http://epp.eurostat.ec.europa.eu/tgm/table.do?tab=table&init=1&plugin=1&language=en&pcode=tec00114

[B23] OECD Stat Extracts http://stats.oecd.org/Index.aspx?DataSetCode=. Accessed June 2013

[B24] Devliegher PJ De Greve A Handicap en armoede in noord en zuid: sociopolitieke factoren en gendercontext 2001 Platform Handicap en Ontwikkelingssamenwerking: Brussel

[B25] Pfaff H Disability and income Wirtschaft und statistic 2005 72 128 134

[B26] Rosano A Mancini F Solipaca A Poverty in people with disabilities: indicators from the capability approach Soc Indic Res 2009 72 75 82 10.1007/s11205-008-9337-1

[B27] Fujiura GT Yamaki K Czechowicz S Disability among ethnic and racial minorities in the United States J Disabil Policy Stud 1998 72 111 130 10.1177/104420739800900207

[B28] Mitra S Findley PA Sambamoorthi U Health care expenditures of living with a disability: total expenditures, out-of-pocket expenses, and burden, 1996 to 2004 Arch Phys Med Rehabil 2009 72 1532 1540 10.1016/j.apmr.2009.02.020 19735781

[B29] Rapalo DM Davis JL Burtner P Bouldin ED Cost as a barrier to dental care among people with disabilities: a report from the Florida behavioral risk factor surveillance system Spec Care Dentist 2010 72 4 133 133 20618778 10.1111/j.1754-4505.2010.00144.x

[B30] Vlaanderen BE Uw wegwijzer binnen de Vlaamse overheid http://www.vlaanderen.be/servlet/Satellite?pagename=nieuwsberichten/NB_KortBestek/Kortbestek&cid=1370462104530&lyt=1106745974281&themaid=1080557605126. Accessed December 2013

[B31] Delafortrie S Springael C Toegankelijkheid van de gezondheidszorg. Presscenter.org http://www.presscenter.org/nl/pressrelease/20130920/toegankelijkheid-van-de-gezondheidszorg. Accessed October 2013

[B32] Federale Overheidsdienst Sociale Zekerheid. Directie-Generaal Personen met een handicap Inkomensvervangende tegemoetkoming stijgt 2% http://www.handicap.fgov.be/nl/nieuws/inkomensvervangende-tegemoetkoming-stijgt-2. Accessed October 2013

[B33] Bossuyt N Gadeyne S Deboosere P Van Oyen H Socio-economic inequalities in health expectancy in Belgium Public Health 2004 72 3 10 10.1016/S0033-3506(03)00130-6 14643622

[B34] Van der Heyden JHA Demarest S Tafforeau J Van Oyen H Socio-economic differences in the utilization of health services in Belgium Health Policy 2003 72 153 165 10.1016/S0168-8510(02)00213-0 12849914

[B35] Van Oyen H Charafeddine R Deboosere P Cox B Lorant V Nusselder W Demarest S Contribution of mortality and disability to the secular trend in health inequality at the turn of century in Belgium Eur J Public Health 2011 72 6 781 787 10.1093/eurpub/ckq198 21217118

[B36] Eurostat. European Commission Income and Living Conditions http://epp.eurostat.ec.europa.eu/portal/page/portal/income_social_inclusion_living_conditions/introduction#. Accessed June 2013

