# Supplementary material for: What socio-demographic factors influence poverty and financial health care access among disabled people in Flanders: a cross-sectional study
Source: Arch Public Health. 2014 Feb 12;72(1):5. doi: 10.1186/2049-3258-72-5 (PMC3930006; doi:10.1186/2049-3258-72-5)
Supplement: Additional file 1 — Description of the population. [file 2049-3258-72-5-S1.docx]

**Appendix: description of the population**

|  |  | N | Percentage  (%) |
| --- | --- | --- | --- |
| Survey |  |  |  |
|  | Paper | 557 | 62.7 |
|  | Online | 332 | 37.3 |
| Gender |  |  |  |
|  | Male | 496 | 55.8 |
|  | Female | 393 | 44.2 |
| Age |  |  |  |
|  | 18-29 | 127 | 14.3 |
|  | 30-39 | 179 | 20.1 |
|  | 40-49 | 241 | 27.1 |
|  | 50-59 | 205 | 23.1 |
|  | 60+ | 137 | 15.4 |
| Type of disability |  |  |  |
|  | Mental | 283 | 31.8 |
|  | Physical | 386 | 43.4 |
|  | Auditory | 33 | 3.7 |
|  | Visual | 66 | 7.4 |
|  | Autism spectrum disorder | 71 | 8.0 |
|  | Psychological | 57 | 6.4 |
|  | Other | 56 | 6.3 |
| Province |  |  |  |
|  | Brussel | 12 | 1.3 |
|  | Vlaams-Brabant | 116 | 13.0 |
|  | Antwerpen | 243 | 27.3 |
|  | Limburg | 92 | 10.3 |
|  | Oost-Vlaanderen | 160 | 18.0 |
|  | West-Vlaanderen | 266 | 29.9 |
| Living alone |  |  |  |
|  | Alone | 518 | 58.3 |
|  | Not alone | 371 | 41.7 |
| Level of dependence |  |  |  |
|  | 1 | 183 | 20.6 |
|  | 2 | 225 | 25.3 |
|  | 3 | 196 | 22.0 |
|  | 4 | 138 | 15.5 |
|  | 5 | 147 | 16.5 |
| Housing |  |  |  |
|  | Institution | 237 | 26.7 |
|  | Private tenant | 189 | 21.3 |
|  | Social tenant | 154 | 17.3 |
|  | Owner | 309 | 34.8 |
| Having a partner |  |  |  |
|  | Yes | 229 | 74.2 |
|  | No | 660 | 25.8 |
| Having children |  |  |  |
|  | At least one | 122 | 13.7 |
|  | None | 767 | 86.3 |
| Number of children |  |  |  |
|  | 0 | 767 | 86.3 |
|  | 1 | 66 | 7.4 |
|  | 2 | 36 | 4.0 |
|  | ≥3 | 20 | 2.2 |
| Employment |  |  |  |
|  | Yes | 208 | 23.4 |
|  | No | 681 | 76.6 |
| Income category |  |  |  |
|  | <€500 | 18 | 2.0 |
|  | €500-€699 | 15 | 1.7 |
|  | €700-€899 | 49 | 5.5 |
|  | €900-€1099 | 158 | 17.8 |
|  | €1100-€1299 | 243 | 27.3 |
|  | €1300-€1499 | 156 | 17.5 |
|  | €1500-€1699 | 55 | 6.2 |
|  | €1700-€1899 | 41 | 4.6 |
|  | €1900-€2099 | 39 | 4.4 |
|  | €2100-€2299 | 28 | 3.1 |
|  | €2300-€2499 | 16 | 1.8 |
|  | €2500-€2699 | 20 | 2.2 |
|  | €2700-€2899 | 7 | 0.8 |
|  | €2900-€3099 | 18 | 2.0 |
|  | €3100-€3299 | 9 | 1.0 |
|  | ≥3300 | 17 | 1.9 |
| Making ends meet |  |  |  |
|  | Not enough | 91 | 10.2 |
|  | Difficult | 382 | 43.0 |
|  | Easy | 394 | 44.3 |
| Satisfaction with income |  |  |  |
|  | Unsatisfied | 305 | 34.3 |
|  | It is enough | 209 | 23.5 |
|  | Satisfied | 367 | 41.3 |
